# Acorenone B: AChE and BChE Inhibitor as a Major Compound of the Essential Oil Distilled from the Ecuadorian Species *Niphogeton dissecta* (Benth.) J.F. Macbr

**DOI:** 10.3390/ph10040084

**Published:** 2017-10-31

**Authors:** James Calva, Nicole Bec, Gianluca Gilardoni, Christian Larroque, Luis Cartuche, Carlo Bicchi, José Vinicio Montesinos

**Affiliations:** 1 Department of Chemistry and Exact Sciences, Private Technical University of Loja (UTPL), Calle Marcelino Champagnat s/n, 1101608 Loja, Ecuador; gianluca.gilardoni@gmail.com (G.G.); lecartuche@utpl.edu.ec (L.C.); jvmontesinos@utpl.edu.ec (J.V.M.); 2 IRCM, Cancer Research Institute of Montpellier, INSERM, U1194, 34298 Montpellier, France; nicole.bec@inserm.fr (N.B.); christian.larroque@inserm.fr (C.L.); 3 Montpellier Regional Cancer Institute, 34298 Montpellier, France; 4 Institut de Recherche en Cancérologie de Montpellier, INSERM, U1194, University of Montpellier, 34298 Montpellier, France; 5 Department of Medicine Science and Technology, University of Turin, Via Pietro Giuria 9, 10125 Turin, Italy; carlo.bicchi@unito.it

**Keywords:** *Niphogeton dissecta*, essential oil, enantiomeric distribution, acorenone B, AChE, BChE

## Abstract

This study investigated the chemical composition, physical proprieties, biological activity, and enantiomeric analysis of the essential oil from the aerial parts of *Niphogeton dissecta* (culantrillo del cerro) from Ecuador, obtained by steam distillation. The qualitative and quantitative analysis of the essential oil was realized by gas chromatographic and spectroscopic techniques (GC-MS and GC-FID). Acorenone B was identified by GC-MS and NMR experiments. The enantiomeric distribution of some constituents has been assessed by enantio-GC through the use of a chiral cyclodextrin-based capillary column. We identified 41 components that accounted for 96.46% of the total analyzed, the major components were acorenone B (41.01%) and (E)-β-ocimene (29.64%). The enantiomeric ratio of (+)/(−)-β-pinene was 86.9:13.1, while the one of (+)/(−)-sabinene was 80.9:19.1. The essential oil showed a weak inhibitory activity, expressed as Minimal Inhibitory Concentration (MIC), against *Enterococcus faecalis* (MIC 10 mg/mL) and *Staphylococcus aureus* (MIC 5 mg/mL). Furthermore, it inhibited butyrylcholinesterase with an IC_50_ value of 11.5 μg/mL. Pure acorenone B showed inhibitory activity against both acetylcholinesterase and butyrylcholinesterase, with IC_50_ values of 40.8 μg/mL and 10.9 μg/mL, respectively.

## 1. Introduction

Medicinal plants have been used for a long time as sources of new pharmaceuticals due to the presence of bioactive compounds [1]. Plants are rich in structurally diverse secondary metabolites displaying a wide range of biological activities, including possible leads for the treatment of neurodegenerative diseases [2,3]. Natural products have contributed greatly as sources for drug discovery for Alzheimer’s disease [4]. Earlier studies have shown that the maintenance of correct levels of acetylcholinesterases is directly related to different diseases such as Alzheimer's disease (AD), bipolar disorder, depression, and schizophrenia [5]. There are two distinct basic types of cholinesterases: acetylcholinesterase (AChE) and butyrylcholinesterase (BChE). The main physiological function of AChE is the splitting of acetylcholine (ACh), a mediator of cholinergic synapses, during the transduction of nerve impulses [6].

AD is a prevalent disease that affects more than 26 million people globally, and is the most common neurodegenerative disease worldwide [7]. AChE inhibitors (AChEIs) have medical applications and are particularly important for the symptomatic treatment of Alzheimer’s disease [8]. The butyrylcholinesterase enzyme (BChE) is synthesized in the liver and its main function is to hydrolyze hydrophobic and hydrophilic carboxylic or phosphoric acid ester containing compounds. Its toxicological and pharmacological importance becomes clear when an individual is exposured to poisonous compounds targeting the acetylcholine binding sites. When there is a hepatic alteration, its concentration decreases in direct relation with the altered hepatocytes [9,10].

The anti-AChE activity of the essential oils of *Eryngium campestre* and *Eryngium amethystine,* two species of the Apiaceae family, is described in the work conducted by Cianfaglione et al. [11], showing that the inhibitory activity is very low (10.5% at 19.5 mg/mL) compared to the commercial inhibitor galanthamine. The activity was expressed as galanthamine equivalent inhibitory activity and the dose tested is similar to an inhibition exerted by 3 μg/mL of the positive control galanthamine. Likewise, the essential oil of *Daucus aristidis* (Apiaceae) tested against AChE and BChE enzymes, by the Ellman method, at a concentration of 100 μg/mL appeared to have a moderate level of inhibitory effect (between 13% to 50%) against both enzymes as compared to that of galanthamine. The moderate AChE inhibitory activity of *Daucus aristidis* essential oil could be explained by the highest concentration of its individual components, α- and β-pinene (over 70%) [12].

Species belonging to the Apiaceae family such as cumin, coriander, carrot, celery, and parsley are considered foods or spices; however, some of them contain highly toxic substances [13]. *Niphogeton dissecta* is a native herb of the Ecuadorian Andes found at 2500–4500 m above sea level [14], and is widely distributed in the provinces of Loja, Azuay, Cañar, Carchi, Chimborazo, Cotopaxi, Morona Santiago, Napo, Pichincha, and Zamora-Chinchipe. Traditionally, it is believed to have medicinal properties and it is used for the treatment of diarrhea, vomiting, inflammation of the belly, colds, and rheumatism [15].

The aim of the present study is to assess the potential antimicrobial, antifungal, and anticholinesterase (AChE and BChE) activity of the essential oil of *Niphogeton dissecta*, as well as to determine its chemical and physical properties. In addition, the phytochemical study of the essential oil led to the isolation and characterization of the known sesquiterpene acorenone B.

## 2. Results and Discussion

The essential oil of the aerial parts of *Niphogeton dissecta* was obtained by steam distillation for 4 h, yielding an average of 0.33 ± 0.03% (*w*/*w*). The physical properties, chemical composition, enantiomeric analysis, and biological activity are discussed below.

### 2.1. Physical Properties

Three physical properties were determined: refractive index (*n* = 1.499 ± 0.002), relative density (*d* = 0.906 ± 0.012 g.m/L), and optical rotation (${\left[\propto \right]}_{D}^{20}=-7.50\pm 0.98$ in CHCl_3_, c = 10.0). In this context, some authors state that the physical properties are determined by the genetic characteristics, geographical location, and phenological stages of the plant [16,17].

### 2.2. Chemical Composition

The chemical composition of the essential oil was defined based on calculated linear retention indices (LRIc) and mass spectra compared with literature [18,19,20,21,22,23]. Table 1 presents the components of the essential oil determined by GC-MS and quantified by GC-FID. Forty-one compounds were separated, which represented 96.46% of the total essential oil. The major compounds were acorenone B (41.01%), (E)-β-ocimene (29.64%), (3E)-butylidene phthalide (5.54%), and α-pinene (3.94%). Oxygenated sesquiterpenes (42.31%) and monoterpene hydrocarbons (37.97%) were the most representative groups. This is the first report on the characterization of the essential oil distilled from *N. dissecta.*

**Figure 1:**
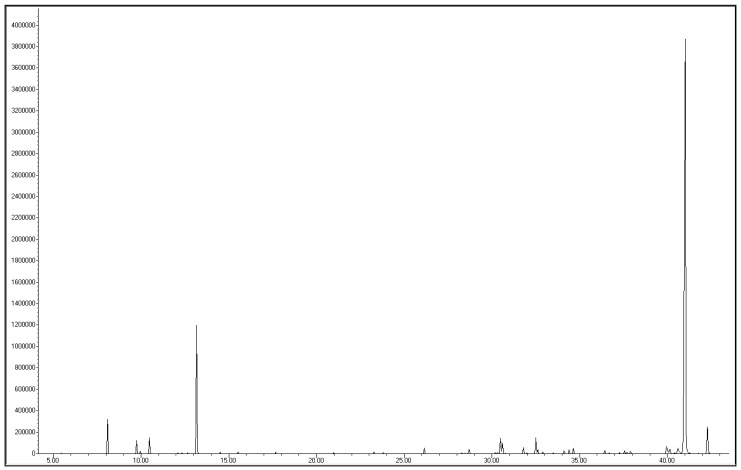
Typical gas-chromatogram of essential oil of *Niphogeton dissecta*.

A typical chromatogram of the essential oil from *N. dissecta* is shown in Figure 1.

### 2.3. Isolation and Characterization of Acorenone B

Acorenone B was isolated by column chromatography on silica gel G60 (1 kg). The mixture (5.0 g) was eluted in isocratic condition with hexane-ethyl acetate 90:10. The compound (1.48 g) was obtained as a pale yellow oil, with optical rotation ${\left[\propto \right]}_{D}^{20}=-17.6$ (CHCl_3_; c = 11.4). Results similar to ours were reported by Zalkow [24].

**Figure 2:**
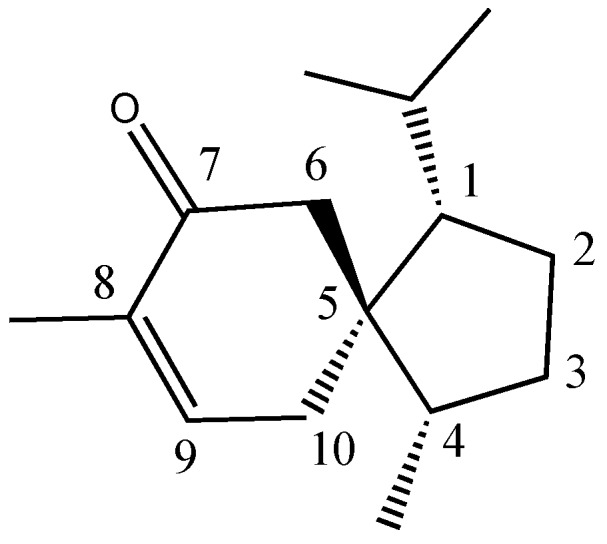
Chemical structure of acorenone B.

The molecular structure of acorenone B (Figure 2) was confirmed by ^1^H NMR, ^13^C NMR, and MS analysis, and compared with data present in the literature [24,25,26,27].

^1^H NMR (400 MHz, CDCl_3_), δ (ppm): 0.74 (3H, d, *J* = 6.8 Hz, CH_3_ of isopropyl), 0.83 (3H, d, *J* = 6.8 Hz, CH_3_ of isopropyl), 0.92 (3H, d, *J* = 6.4 Hz, 4-CH_3_), 1.73 (3H, m, 8-CH_3_), 2.04 (1H, m, H-10), 2.20 (1H, m, H-6) 2.27 (1H, m, H-10), 2.67 (1H, m, H-6), 6.62 (1H, m, H-9).

^13^C NMR (100 MHz, CDCl_3_), δ (ppm): 200.6 (C-7), 144.4 (C-9), 135.4 (C-8), 56.9 (C-1), 49.4 (C-6), 48.4 (C-5), 46.1 (C-4), 29.8 (C-3), 29.2 (C-11), 26.0 (C-10), 25.3 (C-2), 24.2 (C-13), 21.4 (C-14), 17.1 (C-12), 15.6 (C-15).

EI-MS *m*/*z* (%): 220 (M+, 58), 177 (76), 149 (45), 135 (100), 121 (60), 109 (100), 93 (46), 82 (84), 69 (28), 55 (22), 41 (29).

In our study, the fractionation of the essential oil of *Niphogeton dissecta* afforded a pure sequiterpene: acorenone B. Several authors reported the occurrence of acorenone B in other volatile fractions, such as the ones of *Bothriochloa intermedia* (47%) [24], *Bothriochloa pertusa* (9.8%) [28], *Chaerophyllum hirsutum* (9.47% to 18.49%) [26], *Euphorbia macrorrhiza* (16.72% and 25.80%) [29], *Levisticum persicum* (8.3% to 12.6%) [30], and as a major compound in *Daucus littoralis subsp. Hyrcanicus* (19.7% to 57.5% in all parts) [31], and *Bothriochloa bladhii* (18.2%) [32].

The occurrence of acorenone B in *N. dissecta* oil appears somewhat unusual because of the diversity of the sesquiterpenoid skeleton involved. According to Zalkow et al. [24], the proposed structure of acorenone B is considered to be derived from *trans*–*cis*-farnesol via the β-bisabolyl cation.

### 2.4. Enantiomeric Analysis

**Figure 3:**
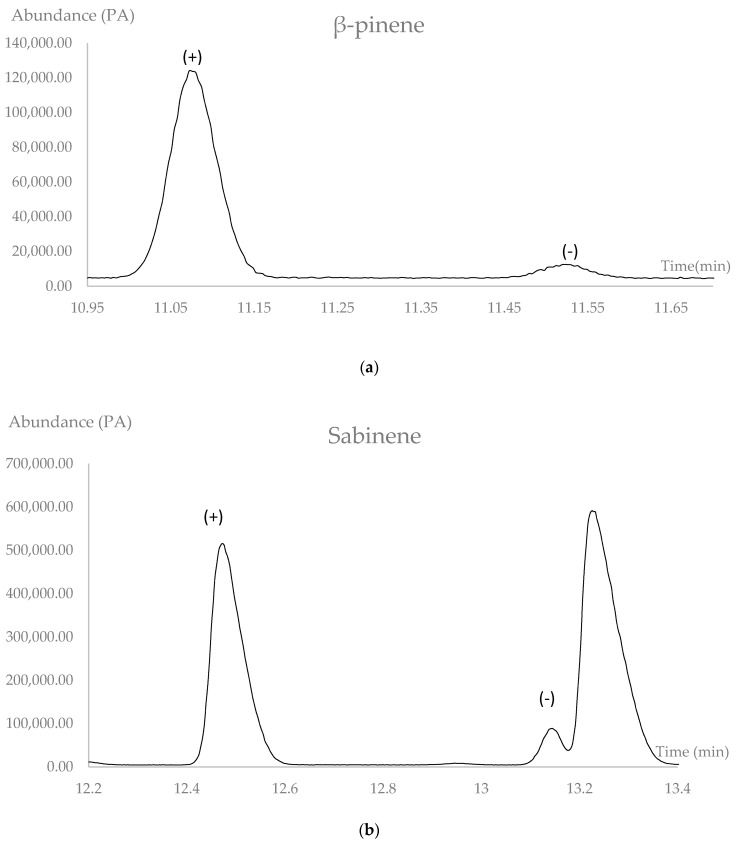
Enantiomeric separation in the essential oil *Niphogeton dissecta*: (**a**) β-pinene; (**b**) sabinene.

The enantiomeric distribution and enantiomeric excess (*e.e.*) (Table 2) of some chiral metabolites were determined on a cyclodextrine-based chiral stationary phase (MEGA-DEX-DET), comparing the retention time of separated enantiomers with enantiomerically pure standards. Two couples of chiral monoterpenoids were detected. The enantiomeric excesses of (+)-β-pinene and (+)-sabinene were quite considerable. These results further confirm that plants can also contain both enantiomers in the essential oil (Figure 3).

It has been documented that sometimes different enantiomers may present dissimilar biological activities [33]. In our essential oil, the enantiomeric excess of (+)-β-pinene was 73.8% with respect to (–)-β-pinene. Some studies have shown that the positive enantiomer has antimicrobial activity against *Candida albicans, Cryptococcus neoformans, Rhyzopus oryzae*, and methicillin-resistant *Staphylococcus aureus* (MRSA) [34]. In contrast, the negative enantiomer exhibits antiviral properties against infectious bronchitis virus (IBV) [35].

### 2.5. Antimicrobial Activity

The essential oil showed a weak inhibitory activity against *Enterococcus faecalis* and *Staphylococcus aureus*, while acorenone B was non-active at the maximum dose tested (10 mg/mL) (Table 3). According to Holetz et al. [36], an antibacterial activity is considered good when the Minimal Inhibitory Concentration (MIC) value is less than 100 μg/mL, demonstrating that both the essential oil and acorenone B do not show inhibitory activity against the evaluated strains.

The weak antimicrobial activity can be explained by the abundance of oxygenated sesquiterpenes in the investigated essential oil. In fact, hydrocarbon sesquiterpenes [29], carvacrol, thymol, eugenol, perylaldehyde, cinnamaldehyde, and cinnamic acid [37] are compounds generally more efficient as antimicrobial inhibitors.

### 2.6. Cholinesterase Inhibition Test

**Figure 4:**
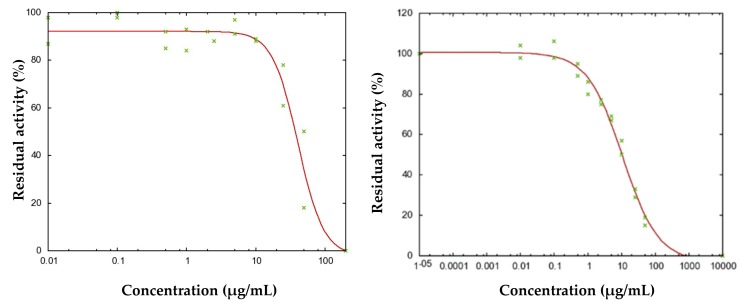
Determination of the IC_50_ values for the Acorenone B vs (**a**) acetylcholinesterase (AChE) and (**b**) butyrylcholinesterase (BChE). IC_50_: half maximal inhibitory concentration.

In the present work, we also evaluated the anti-AChE and anti-BChE activities (Table 4). Acorenone B showed an inhibitory activity against AChE and BChE with IC_50_ concentrations of 40.8 and 10.9 μg/mL, respectively (Figure 4). These inhibitory potentials, even far from that of the reference compound donepezil (6.7 nM) versus AChE [38], are close to those previously published for galanthamine (2.2 µg/mL and 11.7 µg/mL) or other plant extracts [4,39,40]. Interestingly, in spite of its moderate inhibitory potential, galanthamine is a typical drug used for the treatment of Alzheimer's disease [41], validating our efforts to identify new potential inhibitory compounds. *Niphogeton dissecta* essential oil exhibited selectivity for the inhibition of BChE, this property being particularly interesting in the treatment of Alzheimer's disease [40,42].

## 3. Materials and Methods

### 3.1. Plant Material

The aerial parts of *Niphogeton dissecta* in flowering state were collected in September and October 2016 in the sector Loma la Torre, Loja, at an altitude of 3210 m a.s.l., with coordinates 696030 N, 9593252 E. The specimen was identified by Dr. Fani Tinitana of the UTPL herbarium, and deposited with voucher number 5835. The plant was collected under permission of the Ministry of Environment of Ecuador (MAE-DNB-CM-2016-0048).

### 3.2. Extraction of Essential Oil

The essential oil was obtained from the aerial parts, by steam distillation in a Clevenger-type apparatus for 4 h. Four distillations were carried out with 1805, 751, 794, and 758 grams of fresh plant material, respectively. The oil was dried on anhydrous sodium sulfate and then stored at −14 °C. The yield was expressed as mean values and standard deviations of the four distillations and reported as percentages of *w*/*w*.

### 3.3. Physical Analysis

The relativity density (*d*^20^) was determined using a 1-cm^3^ pycnometer. The refractive index (*n*^20^) was measured by an Abbe's refractometer, manufactured by Boeco, Germany. The specific optical rotation ${\left[\propto \right]}_{D}^{20}$ was determined in a Hanon P 810 automatic polarimeter. All these properties were expressed as mean values and standard deviations of four measurements.

### 3.4. Gas Chromatography Coupled to Mass-Spectrometry (GC-MS)

The chemical constituents of the *Niphogeton dissecta* essential oil were analyzed on an Agilent Technologies 6890N gas chromatograph, coupled to a 5973N mass spectrometer (Santa Clara, CA, USA) and equipped with a DB-5MS capillary column (5%-phenyl-methylpolysiloxane, 30 m, 0.25 mm internal diameter., 0.25 μm film thickness; J & W Scientific, Folsom, CA, USA). For the separation of the volatile constituents, the following temperature program was used: 5 min at 60 °C, 3 °C/min up to 165 °C, 15 °C/min up to 250 °C, and held for 10 min. The injector and detector temperatures were kept at 220 °C. The carrier gas was helium, at a flow rate of 1 mL/min. The injector was operated in split mode, with a split ratio of 1:50. The acquisition mass range was set at 40–350 *m*/*z*. Ionization mode: electron-impact (70 eV). The essential oil was diluted 1:100 *v*/*v* in dichloromethane (Fisher Scientific, 99.9% purity) and 1 µL of the solution was injected.

For the identification of the essential oil components, linear retention indices were calculated according to Van Den Dool and Kratz. They were determined with a homologous series of linear alkanes (C9 from BDH, purity 99%, and C10–C25 from Fluka, purity 99%).

### 3.5. Gas Chromatography Coupled to Flame Ionization Detector (GC-FID)

Quantitative analysis of the essential oil was performed on an Agilent Technologies gas chromatograph (model 6890N) coupled to a flame ionization detector (FID) and using a 7683 series autoinjector (Agilent, Little Falls, DE, USA). The percentage composition of the oil was determined by correlating GC peak areas to the total chromatogram, without applying any correction factor, but normalizing values with nonane as an internal standard. The qualitative analysis is expressed as the mean values of four injections and standard deviations. The analytical parameters were the same as the GC-MS analysis.

### 3.6. Enantioselective GC Analysis

Enantioselective GC-MS analysis was performed with the same Agilent Technologies instrument previously described. The mass spectrometer operated in electron impact ionization mode at 70 eV, with a mass range of *m*/*z* 40–350 full scan mode. The ion source temperature was set at 220 °C. Helium was the carrier gas at a flow rate of 1.0 mL/min. The injector was operated in split mode (1:40) at 200 °C, with the transfer line at 230 °C. The oven thermal program was as follows: 60 °C for 2 min, then the temperature was raised to 220 °C with a gradient rate of 2 °C/min and held at 220 °C for 2 min. A chiral capillary column based on diethyl tertbutylsilyl-BETA-cyclodextrin (25m × 0.25mm × film thickness 0.25 μm) from Mega (Legnano, MI, Italy) was used. The essential oil was diluted 5:100 (*v*/*v*) in dichloromethane (Fisher Scientific, 99.9% purity) and 1 µL of the solution was injected. Enantiomerically pure standards, used to determine the elution order of enantiomers, were available in one of the authors' laboratory (C.B.).

### 3.7. Isolation and Identification of Acorenone B

The essential oil of *N. dissecta* (5 g) was subjected to column chromatography over a silica gel G60 column, applying an oil/silica weight ratio of 1:200, with a mixture of Hex:AcOEt 90:10 (isocratic elution), obtaining a total of five fractions and affording acorenone B as a pure compound (1.48 g). The identification was performed by spectroscopic techniques such as EI-MS, ^1^H, and ^13^C NMR. ^1^H and ^13^C NMR spectra were acquired using a VARIAN NMR spectrometer (400 MHz for ^1^H and 100 MHz for ^13^C), tetramethylsilane was used as an internal standard, and chemical shifts are given in δ (ppm).

### 3.8. Antimicrobial Activity

Five pathogenic bacteria (ATCC): *Staphylococcus aureus* ATCC 25923, *E. faecalis* ATCC 29212, *Pseudomonas aeruginosa* ATCC 27853, *Escherichia coli* ATCC 25922, *Proteus vulgaris* ATCC 8427, and *Klebsiella pneumoniae* ATCC 9997 were included in the investigation. For all bacteria, except for *E. faecalis*, a heart-heart infusion broth (BHI-DIFCO, DIFCO, Sparks, MD, USA) was used. All the strains were maintained at −80 °C until use, when they were withdrawn to prepare overnight cultures at 37 °C for 16 h. MIC values were determined by the micro-dilution broth method, using a final concentration of 5/105 CFU/mL. DMSO solutions of the sample were prepared at a concentration of 20 mg/mL. Assays were carried out in 96-well plates and a two-fold serial dilution was used to obtain decreasing concentrations from 1000 to 0.024 mg/mL. The incubation was performed at 37 °C for 24 h. Gentamicin was used a positive control with an MIC value of 0.40 mg/mL, except for *E. faecalis* where ampicillin (MIC 1.56 mg/mL) was used.

### 3.9. Cholinesterase Inhibition Test

The cholinesterase (ChE) activities were assayed following a colorimetric protocol adapted from Ellman et al. [43,44]. ChEs efficiently catalyze the hydrolysis of acetylthiocholine (ATCh), the sulfur analog of the natural substrate of these enzymes. Upon hydrolysis, this substrate analog produces acetate ion and thiocholine. Thiocholine, in the presence of the highly reactive dithiobisnitrobenzoate (DTNB) ion, generates a yellow color, which can be quantitatively monitored by spectrophotometric absorption at 412 nm. All reagents were obtained from the Sigma-Aldrich trading house. A typical 200 μL inhibition assay volume contained phosphate buffered saline solution (pH 7.4), DTNB (1.5 mM), test sample in DMSO (1% *v/v* final). Both acetylcholinesterase from *Electrophorus electricus* (Type V-S, lyophilized powder, 744 U/mg solid, 1 272 U/mg protein) and butyrylcholinesterase from equine serum (lyophilized powder, ≥900 units/mg protein) were dissolved in PBS pH 7.4 and used at 25 mU/mL for the assay. After 10 min of pre-incubation, the substrate acetylthiocholine iodide (1.5 mM) was added to start the reaction. During 1 h of incubation at 30 °C, 96-well microtiter multiplates were read on a PherastarFS (BMG Labtech) detection system. All measurements were made in triplicate. When possible, the IC_50_ values were calculated using the GNUPLOT package on line (www.ic50.tk, www.gnuplot.info). Donepezil was used as reference ChE inhibitor with an IC_50_ = 100 nM for AChE and 8500 nM for BChE. In this assay, we did not exclude the possibility of false-positive inhibition results previously described for high concentrations (>100 μg/mL) of amine or aldehyde compounds [45,46], but the lack of inhibition observed for the essential oil versus the AChE strongly minimized this possibility.

## 4. Conclusions

The physical properties, chemical composition, biological activity, and enantiomeric distribution of the essential oil distilled from *Niphogeton dissecta* were determined for the first time. Forty-one compounds, representing 96.46% of the total oil, were identified. The major compounds were acorenone B (41.01%), (E)-β-ocimene (29.64%), (3E)-butylidene phthalide (5.54%), and α-pinene (3.94%). The whole volatile fraction and its pure major constituent were assayed for two biological activities: inhibition of two bacteria strains and enzymatic inhibition against AChE and BChE. Concerning the first activity, the oil showed an MIC value of 10 mg/mL against *Enterococcus faecalis* and 5 mg/mL against *Staphylococcus aureus*. No activity was detected for pure acorenone B. Concerning the enzymatic inhibition, the Essential Oil (E.O.) showed an IC_50_ value of 11.5 μg/mL in the inhibition of BChE and no inhibition of AChE. On the other hand, acorenone B was active against both enzymes, with an IC_50_ of 40.8 μg/mL for AChE and 10.9 μg/mL for BChE. The chiral analysis of the E.O. indicated the following enantiomeric distribution: (+)-β-pinene (86.9%), (−)-β-pinene (13.1%), (+)-sabinene (80.9%), and (−)-sabinene (19.1%).

## Figures and Tables

**Table 1:** Chemical composition of *Niphogeton dissecta* essential oil of province of Loja, Ecuador.

Component	LRI ^a^	LRI lit ^b^	% ^c^	δ	Literature for LRI
α-Pinene	930	932	3.94	1.79	[18]
Sabinene	969	969	1.41	0.28	[18]
β-Pinene	974	974	0.30	0.09	[18]
β-Myrcene	987	988	2.14	0.37	[18]
p-Cymene	1022	1020	trace	--	[18]
Limonene	1027	1024	0.13	0.03	[18]
(Z)-β-Ocimene	1034	1032	0.10	0.04	[18]
(E)-β-Ocimene	1045	1044	29.64	1.63	[18]
γ-Terpinolene	1055	1056	0.29	0.30	[19]
Unidentified	1072	--	trace	--	--
α-Pinene oxide	1093	1099	trace	--	[18]
Unidentified	1155	--	trace	--	--
(E,E) 2,6-Dimethyl-3,5,7-octatriene-2-ol	1205	1207	trace	--	[20]
Citronellol	1224	1223	0.16	0.07	[18]
Unidentified	1256	--	0.15	0.01	--
Geranial	1264	1264	0.07	0.01	[18]
Methyl geranate	1318	1322	0.51	0.04	[18]
Unidentified	1334	--	0.06	0.01	--
α-Copaene	1371	1374	trace	--	[18]
β-Funebrene	1411	1413	0.17	0.02	[18]
(E)-Caryophyllene	1414	1417	0.24	0.02	[18]
β-Cedrene	1417	1419	1.23	0.13	[18]
cis-Thujopsene	1420	1429	0.97	0.10	[18]
(E)-β-Farnesene	1450	1454	1.13	0.13	[18]
allo-aromadendrene	1454	1458	trace	--	[18]
α-Himachalene	1457	1449	trace	--	[18]
cis-Cadina-1(6),4-diene	1466	1461	1.70	0.17	[18]
γ-Muurolene	1469	1478	0.36	0.04	[18]
Germacrene-D	1475	1484	0.38	0.05	[18]
ar-Curcumene	1477	1479	0.15	0.01	[18]
α-Zingiberene	1491	1493	0.34	0.02	[18]
(E,E)-α-Farnesene	1501	1505	trace	--	[18]
Unidentified	1506	--	0.23	0.02	--
δ-Cadinene	1513	1522	0.36	0.03	[18]
β-Sesquiphellandrene	1519	1521	0.89	0.08	[18]
(E)-Nerolidol	1557	1561	0.08	0.01	[18]
Unidentified	1565	--	0.42	0.07	--
Spathulenol	1569	1577	0.13	0.01	[18]
Unidentified	1581	--	0.40	0.03	--
Unidentified	1585	--	0.15	0.01	--
Geranyl isovalerate	1594	1606	0.57	0.05	[18]
Cedrol	1600	1600	0.33	0.03	[18]
Unidentified	1609	--	0.07	0.01	--
α-Cadinol	1645	1652	0.08	0.01	[18]
Unidentified	1655	--	1.20	0.05	--
Acorenone	1666	1655	0.11	0.01	[21]
Acorenone B	1683	1675	41.01	3.35	[22]
(3E)-Butylidene phthalide	1718	1717	5.54	0.93	[18]
Unidentified	1777	--	0.11	0.04	--
Sandaracopimaradiene	1943	1942	trace	--	[23]
Biformene	1983	1990	trace	--	[18]
Unidentified	2008	--	trace	--	--
(E,E)-Geranyl linalool	2016	2026	trace	--	[18]
Unidentified	2047	--	trace	--	--
Unidentified	2052	--	0.64	0.19	--
Monoterpene hydrocarbons	-	-	37.97		
Oxygenated monoterpene	-	-	0.25		
Sesquiterpene hydrocarbons	-	-	9.71		
Oxygenated sesquiterpene	-	-	42.31		
Others	-	-	6.22		
Total amount of compounds	-	-	96.46%		

**^a^** Calculated linear retention indices (LRI) on DB-5MS capillary column; **^b^** Linear retention indices according to literature; **^c^** Relative percentage values are means of four determinations with a Relative Standard Deviation (RSD%) below 5% for the most abundant components. Traces % < 0.05.

**Table 2:** Enantiomeric excess of some essential oil constituents from *Niphogeton dissecta*.

Components	RT ^a^ (min)	LRI ^b^	Enantiomeric Distribution (%)	*e.e.* (%)
(+)-β-pinene	11.08	957	86.9	73.8
(−)-β-pinene	11.52	965	13.1	
(+)-Sabinene	12.47	983	80.9	61.8
(−)-Sabinene	13.15	997	19.1	

**^a^** Retention Time (RT); **^b^** Calculated on MEGA-DEX-DET chiral stationary phase.

**Table 3:** Strains used for biological tests.

Microorganism	E.O, (*Niphogeton dissecta*) mg/mL	Acorenone B (mg/mL)
*Candida albicans*	NA	NA
*Enterococcus faecalis*	10	NA
*Escherichia coli*	NA	NA
*Micrococcus luteus*	NA	NA
*Staphylococcus aureus*	5	NA

NA = non-active.

**Table 4:** Cholinesterase inhibitory activity.

Compound	AChE (IC_50_) μg/mL	BChE (IC_50_) μg/mL
*Niphogeton dissecta* essential oil	NA	11.5
Acorenone B	40.8	10.9

NA = non-active.

## References

[1] Panda S.K., Mohanta Y.K., Padhi L., Park Y.H., Mohanta T.K., Bae H. (2016). Large scale screening of ethnomedicinal plants for identification of potential antibacterial compounds. Molecules.

[2] Christensen L.P., Brandt K. (2006). Bioactive polyacetylenes in food plants of the Apiaceae family: Occurrence, bioactivity and analysis. J. Pharm. Biomed. Anal..

[3] Hanafy D.M., Prenzler P.D., Burrows G.E., Ryan D., Nielsen S., El Sawi S.A., Obied H.K. (2017). Biophenols of mints: Antioxidant, acetylcholinesterase, butyrylcholinesterase and histone deacetylase inhibition activities targeting Alzheimer’s disease treatment. J. Funct. Foods.

[4] Fadaeinasab M., Hadi A.H.A., Kia Y., Basiri A., Murugaiyah V. (2013). Cholinesterase enzymes inhibitors from the leaves of Rauvolfia reflexa and their molecular docking study. Molecules.

[5] Zachow L.L., Ávila J.M., Saldanha G.A., Mostardeiro M.A., da Silva U.F., Morel A.F., Dalcol I.I. (2017). Chemical composition and evaluation of prolyl oligopeptidase and acetylcholinesterase inhibitory activities of Leonurus Sibiricus L. from Brazil. Nat. Prod. Res..

[6] Chuiko G., Podgornaya V., Zhelnin Y. (2003). Acetylcholinesterase and butyrylcholinesterase activities in brain and plasma of freshwater teleosts: Cross-species and cross-family differences. Comp. Biochem. Physiol. Part B Biochem. Mol. Biol..

[7] Manalo R.V., Silvestre M.A., Barbosa A.L.A., Medina P.M. (2017). Coconut (*Cocos nucifera*) Ethanolic Leaf Extract Reduces Amyloid-β (1–42) Aggregation and Paralysis Prevalence in Transgenic Caenorhabditis elegans Independently of Free Radical Scavenging and Acetylcholinesterase Inhibition. Biomedicines.

[8] Akıncıoğlu A., Akıncıoğlu H., Gülçin İ., Durdagi S., Supuran C.T., Göksu S. (2015). Discovery of potent carbonic anhydrase and acetylcholine esterase inhibitors: Novel sulfamoylcarbamates and sulfamides derived from acetophenones. Bioorg. Med. Chem..

[9] Vásquez L., Osorio J. (2000). Variación de la actividad de la enzima Butirilcolinesterasa en usuarias de anticonceptivos hormonales. Anales de la Facultad de Medicina.

[10] Çokuğraş A.N. (2003). Butyrylcholinesterase: structure and physiological importance. Turk. J. Biochem..

[11] Cianfaglione K., Blomme E.E., Quassinti L., Bramucci M., Lupidi G., Dall’Acqua S., Maggi F. (2017). Cytotoxic Essential Oils from Eryngium campestre and Eryngium amethystinum (Apiaceae) Growing in Central Italy. Chem. Biodivers..

[12] Lamamra M., Laouer H., Amira S., Orhan I.E., Senol F.S., Demirci B., Akkal S. (2017). Chemical Composition and Cholinesterase Inhibitory Activity of Different Parts of Daucus aristidis Coss. Essential Oils from Two Locations in Algeria. Rec. Natl. Prod..

[13] Baldemir A., Topçu H., Paksoy M.Y., Motalebipour E.Z., Kafkas S. (2017). First microsatellite markers for Scaligeria lazica Boiss.(Apiaceae) by next-generation sequencing: Population structure and genetic diversity analysis. Biotechnol. Biotechnol. Equip..

[14] Izco J., Pulgar Í., Aguirre Z., Santin F. (2007). Estudio florístico de los páramos de pajonal meridionales de Ecuador. Rev. Peru. Biol..

[15] Abad X. (2009). Determinación de la Actividad Antimicrobiana de los Extractos Totales de Cuatro Especies Vegetales de las Provincias de Loja y Zamora Chinchipe: Piper ecuadorense (Matico), Lepechinia mutica Benth (Turuyante), Fuschia ayavacensis (Pena-Pena), Niphogeton dissecta (Culantrillo del cerro), Empleando los Métodos de Difusión en Placa, Concentración Mínima Inhibitoria e Inhibición del Crecimiento Radial.

[16] Rajabi Z., Ebrahimi M., Farajpour M., Mirza M., Ramshini H. (2014). Compositions and yield variation of essential oils among and within nine Salvia species from various areas of Iran. Ind. Crops Prod..

[17] Benyelles B., Allali H., Dib M.E.A., Djabou N., Paolini J., Costa J. (2017). Chemical Composition Variability of Essential Oils of *Daucus gracilis* Steinh. from Algeria. Chem. Biodivers..

[18] Adams R.P. (2009). Identification of Essential Oil Components by Gas Chromatography/Mass Spectrometry.

[19] Angioni A., Barra A., Coroneo V., Dessi S., Cabras P. (2006). Chemical composition, seasonal variability, and antifungal activity of *Lavandula stoechas* L. ssp. stoechas essential oils from stem/leaves and flowers. J. Agric. Food Chem..

[20] Jalali-Heravi M., Zekavat B., Sereshti H. (2006). Characterization of essential oil components of Iranian geranium oil using gas chromatography–mass spectrometry combined with chemometric resolution techniques. J. Chromatogr. A.

[21] Marongiu B., Piras A., Porcedda S., Scorciapino A. (2005). Chemical composition of the essential oil and supercritical CO_2_ extract of *Commiphora myrrha* (Nees) Engl. and of *Acorus calamus* L.. J. Agric. Food Chem..

[22] Hammami I., Triki M.A., Rebai A. (2011). Chemical compositions, antibacterial and antioxidant activities of essential oil and various extracts of *Geranium sanguineum* L. flowers. Arch. Appl. Sci. Res..

[23] Skaltsa H.D., Mavrommati A., Constantinidis T. (2001). A chemotaxonomic investigation of volatile constituents in Stachys subsect. Swainsonianeae (Labiatae). Phytochemistry.

[24] Zalkow L.H., Baxter J.T., McClure R.J., Gordon M.M. (1980). A Phytochemical Investigation of Bothriochloa intermedia. J. Natl. Prod..

[25] Yousefbeyk F., Golfakhrabadi F., Amouei A., Ghasemi S., Amin M., Gohari A., Samadi N., Amini M., Amin G. (2015). Phytochemical Investigation and Antifungal Activity of *Daucus littoralis* Smith sub sp. hyrcanicus Rech. f.. Res. J. Phytochem..

[26] Kubeczka K.-H., Bohn I., Schultze W., Formaček V. (1989). The Composition of the Essential Oils of *Chaerophyllum hirsutum* L.. J. Essent. Oil Res..

[27] Lawrence B.M., Morton J.K. (1974). Acorenone-B in Angelica lucida oil. Phytochemistry.

[28] Kaul V.K., Vats S. (1998). Essential oil composition of *Bothriochloa pertusa* and phyletic relationship in aromatic grasses. Biochem. Syst. Ecol..

[29] Lin J., Dou J., Xu J., Aisa H.A. (2012). Chemical composition, antimicrobial and antitumor activities of the essential oils and crude extracts of Euphorbia macrorrhiza. Molecules.

[30] Shafaghat A. (2011). Chemical constituents, antimicrobial and antioxidant activity of the hexane extract from root and seed of *Levisticum persicum* Freyn and Bornm. J. Med. Plants Res..

[31] Yousefbeyk F., Gohari A.R., Sourmaghi M.H.S., Amini M., Jamalifar H., Amin M., Golfakhrabadi F., Ramezani N., Amin G. (2014). Chemical Composition and Antimicrobial Activity of Essential Oils from Different Parts of *Daucus littoralis* Smith subsp. *hyrcanicus* Rech. f.. J. Essent. Oil Bear. Plants.

[32] Bahl J., Padalia R.C., Verma R.S., Bansal R.P. (2014). Essential Oil Composition of *Bothriochloa bladhii* (Retz.) ST Blake: An Introduction from Tropical Region of Western Ghats of India. J. Essent. Oil Bear. Plants.

[33] Pérez-Fernández V., García M.Á., Marina M.L. (2010). Characteristics and enantiomeric analysis of chiral pyrethroids. J. Chromatogr. A.

[34] Silva A.C.R.D., Lopes P.M., Azevedo M.M.B.D., Costa D.C.M., Alviano C.S., Alviano D.S. (2012). Biological activities of a-pinene and β-pinene enantiomers. Molecules.

[35] Yang Z., Wu N., Zu Y., Fu Y. (2011). Comparative Anti-Infectious Bronchitis Virus (IBV) Activity of (−)-Pinene: Effect on Nucleocapsid (N) Protein. Molecules.

[36] Holetz F.B., Pessini G.L., Sanches N.R., Cortez D.A.G., Nakamura C.V., Dias Filho B.P. (2002). Screening of some plants used in the Brazilian folk medicine for the treatment of infectious diseases. Memórias Instituto Oswaldo Cruz.

[37] Burt S. (2004). Essential oils: Their antibacterial properties and potential applications in foods—A review. Int. J. Food Microbiol..

[38] Ogura H., Kosasa T., Kuriya Y., Yamanishi Y. (2000). Comparison of inhibitory activities of donepezil and other cholinesterase inhibitors on acetylcholinesterase and butyrylcholinesterase in vitro. Methods Find. Exp. Clin. Pharmacol..

[39] Costa P., Grosso C., Gonçalves S., Andrade P.B., Valentão P., Bernardo-Gil M.G., Romano A. (2012). Supercritical fluid extraction and hydrodistillation for the recovery of bioactive compounds from Lavandula viridis L’Hér. Food Chem..

[40] Zeb A., Hameed A., Khan L., Khan I., Dalvandi K., Iqbal Choudhary M., Basha F.Z. (2014). Quinoxaline derivatives: Novel and selective butyrylcholinesterase inhibitors. Med. Chem..

[41] Bloniecki V., Aarsland D., Blennow K., Cummings J., Falahati F., Winblad B., Freund-Levi Y. (2017). Effects of Risperidone and Galantamine Treatment on Alzheimer’s Disease Biomarker Levels in Cerebrospinal Fluid. J. Alzheimer's Dis..

[42] Greig N.H., Utsuki T., Ingram D.K., Wang Y., Pepeu G., Scali C., Yu Q.S., Mamczarz J., Holloway H.W., Giordano T. (2005). Selective butyrylcholinesterase inhibition elevates brain acetylcholine, augments learning and lowers Alzheimer β-amyloid peptide in rodent. Proc. Natl. Acad. Sci. USA.

[43] Ellman G.L., Courtney K.D., Andres V., Featherstone R.M. (1961). A new and rapid colorimetric determination of acetylcholinesterase activity. Biochem. Pharmacol..

[44] Armijos C., Gilardoni G., Amay L., Lozano A., Bracco F., Ramirez J., Bec N., Larroque C., Vita Finzi P.V., Vidari G. (2016). Phytochemical and ethnomedicinal study of Huperzia species used in the traditional medicine of Saraguros in Southern Ecuador; AChE and MAO inhibitory activity. J. Ethnopharmacol..

[45] Rhee I.K., Van Rijn R.M., Verpoorte R. (2003). Qualitative determination of false positive effects in the acetylcholinesterase assays using thin layer chromatography. Phytochem. Anal..

[46] Rhee I.K., van de Meent M., Ingkaninan K., Verpoorte R. (2001). Screening for acetylcholinesterase inhibitors from Amaryllidaceae using silica gel thin-layer chromatography in combination with bioactivity staining. J. Chromatogr. A.

